# Multimodality Imaging in Cardiooncology

**DOI:** 10.1155/2015/263950

**Published:** 2015-08-02

**Authors:** Fausto Pizzino, Giampiero Vizzari, Rubina Qamar, Charles Bomzer, Scipione Carerj, Concetta Zito, Bijoy K. Khandheria

**Affiliations:** ^1^Cardiology Unit, Department of Clinical and Experimental Medicine, University of Messina, Azienda Ospedaliera Universitaria “Policlinico G. Martino” and Universita' degli Studi di Messina, Via Consolare Valeria No. 12, 98100 Messina, Italy; ^2^Aurora Advanced Healthcare, St. Luke's Medical Centers, 2801 W. Kinnickinnic River Parkway, No. 840, Milwaukee, WI 53215, USA; ^3^Aurora Cardiovascular Services, Aurora Sinai/Aurora St. Luke's Medical Centers, University of Wisconsin School of Medicine and Public Health, 2801 W. Kinnickinnic River Parkway, No. 840, Milwaukee, WI 53215, USA

## Abstract

Cardiotoxicity represents a rising problem influencing prognosis and quality of life of chemotherapy-treated patients. Anthracyclines and trastuzumab are the drugs most commonly associated with development of a cardiotoxic effect. Heart failure, myocardial ischemia, hypertension, myocarditis, and thrombosis are typical manifestation of cardiotoxicity by chemotherapeutic agents. Diagnosis and monitoring of cardiac side-effects of cancer treatment is of paramount importance. Echocardiography and nuclear medicine methods are widely used in clinical practice and left ventricular ejection fraction is the most important parameter to asses myocardial damage secondary to chemotherapy. However, left ventricular ejection decrease is a delayed phenomenon, occurring after a long stage of silent myocardial damage that classic imaging methods are not able to detect. New imaging techniques including three-dimensional echocardiography, speckle tracking echocardiography, and cardiac magnetic resonance have demonstrated high sensitivity in detecting the earliest alteration of left ventricular function associated with future development of chemotherapy-induced cardiomyopathy. Early diagnosis of cardiac involvement in cancer patients can allow for timely and adequate treatment management and the introduction of cardioprotective strategies.

## 1. Introduction

Chemotherapy is widely used in the treatment of several neoplastic diseases, leading to an improvement in survival and prognosis in a large number of patients. Side effects are the most common cause of restriction to its use. Cardiotoxicity represents a frequent complication secondary to the intake of some classes of chemotherapeutic agents, with significant consequences on patients' outcome [1]. Heart failure (HF) is the most common manifestation of chemotherapy induced cardiotoxicity. Although left ventricular ejection fraction (LVEF) is widely utilized in monitoring the cardiac function in clinical practice, it has not demonstrated high sensitivity in detecting subclinical myocardial dysfunction. New parameters and new imaging techniques have been developed in order to overcome the limitations related to isolate evaluation of LVEF [2, 3]. A diagnostic approach based on the integrative use of different imaging techniques can allow early detection of cardiotoxicity, improving the therapeutic management of the neoplastic disease, quality of life, and mortality rate.

## 2. Clinical Manifestations of Cardiotoxicity

HF occurs with an incidence range included between 0.5 and 28%, depending on the medication used, and is the most common clinical manifestation of the cardiotoxicity induced by chemotherapy [1]. The onset of dyspnea, chest pain, peripheral edema, and asthenia is usually preceded by a variable stage of subclinical myocardial dysfunction. Traditionally cardiotoxicity induced by chemotherapy has been classified into two groups [4]: Type I chemotherapy-related myocardial dysfunction is typical of anthracyclines and has been related to oxidative stress causing myocardiocytes damage and death; it is an irreversible, dose-dependent process and is characterized by ultrastructural alteration identifiable by myocardial biopsy. Type II chemotherapy-related myocardial dysfunction is induced by trastuzumab and is related to the inhibition of ErbB2 pathway. Usually the dysfunction is reversible and not related to the cumulative dose [5].

Coronary artery disease, presenting with asymptomatic T-wave changes, chest pain, acute coronary syndromes, and myocardial infarction, is mainly related to use of antimetabolites (particularly 5-fluorouracil). De Forni reported an incidence of acute coronary syndromes of about 7.6% in patients treated with 5-fluorouracil while cardiac mortality reached 2.2% [6].

Hypertension is a relatively common side effect of several antiangiogenetic drugs like bevacizumab, sunitinib, and sorafenib. Underlying artery hypertension is the most important risk factor for the development of the secondary disease.

Cancer patients have a high incidence of thromboembolic events depending on cancer-related factors (primitive malignancy localization, immobility, HF, arrhythmias, etc.) [7] and additional effects of some chemotherapeutic agents, particularly, cisplatin and thalidomide [8, 9].

## 3. Cancer Treatment and Cardiotoxicity: Who Are the Actors?

The majority of studies on cardiotoxicity focus on patients treated with anthracyclines and trastuzumab. Anthracyclines (doxorubicin, daunorubicin, and epirubicin) use has been related to onset of HF within 1 year in about 2% of treated patients [1]. The HF incidence increases to 28% when the patients are exposed to the association of anthracyclines and trastuzumab [1]. Cardiotoxic effect has been described for classes of drugs other than the anthracyclines and trastuzumab such as inhibitors of tyrosine kinases (imatinib, dasatinib, nilotinib, sunitinib, sorafenib, and bevacizumab), antimetabolites (5-fluorouracil), alkylating agents (cisplatin, cyclophosphamide), and taxanes (docetaxel and paclitaxel) [10]. Radiotherapy has become an important instrument in the treatment of several malignances and is more often associated to standard chemotherapy treatment. Irradiation of the mediastinum with a cumulative dose >30 Gy and a daily fractioning >2 Gy appeared to be related to a high risk of developing cardiac dysfunction [11].

## 4. How to Diagnose Cardiotoxicity? The Need for Multimodality Imaging

Myocardial biopsy is still considered the most accurate and specific method in identifying the myocardial damage induced by chemotherapy, detecting the ultrastructural alteration of cardiomyocytes [12]. Nevertheless its invasiveness limited its use in clinical practice. Imaging methods emerged in the last decades as the landmark in monitoring cardiotoxicity in cancer patients. Left ventricular ejection fraction (LVEF) is widely considered the most important parameter for the diagnosis of cardiotoxicity. The most validated definition of cardiotoxicity has been established by the cardiac review and evaluation committee [13]. Cardiotoxicity can be defined either by the onset of HF symptoms and signs or by an asymptomatic decrease of LVEF as follows.

*Cardiac Review and Evaluation Committee Criteria for Diagnosis of Cardiotoxicity*. The diagnosis of cardiotoxicity is established if one or more criteria are present:

 cardiomyopathy characterized by a decrease in cardiac LVEF that was either global or more severe in the septum,
 symptoms of congestive heart failure,
 associated signs of congestive heart failure, including but not limited to third heart sound (S3) gallop, tachycardia, or both,
 decline in LVEF of at least 5% to less than 55% with accompanying signs or symptoms of congestive heart failure,
 decline in LVEF of at least 10% to below 55% without accompanying signs or symptoms.

Although the evolution of most recent imaging techniques has allowed accurate and reproducible evaluation of volumes and of alteration of LVEF, recently it has appeared evident that the drop of LVEF represents a late phenomenon in the physiopathology of the chemotherapy-induced cardiotoxicity. This evidence has led the clinicians to look to other imaging methods that evaluate cardiac function independently of cardiac volumes changes, aiming to detect the earliest manifestation of cardiotoxicity and allowing for the appropriate management of the therapy. Some of these methods such as speckle tracking imaging have been already introduced in clinical practice whereas others are under investigation in experimental settings.

## 5. Methods Based on the Evaluation of LVEF: From Echocardiography to Cardiac Magnetic Resonance

### 5.1. Two-Dimensional Echocardiography

LVEF evaluated by two-dimensional echocardiography (2DE) is the most used parameter in monitoring the cardiac function in chemotherapy-treated patients (Videos 1 and 2; see Supplementary Material available online at http://dx.doi.org/10.1155/2015/263950). The Simpson biplane method is the most validated technique to obtain the left ventricle volumes, while monodimensional measurements are less accurate. However, LVEF derived by the Simpson formula relies on geometrical assumptions and the manual tracking of the endocardial border can differ when performed by different observers, particularly with poor quality images. Indeed, a recent investigation reported that 2DE is unable to estimate a decrease <10% within the 95% of confidence interval when performed by different investigators [14]; considering that cardiotoxicity has been defined as a drop of LVEF ≥10% or ≥5% in presence of HF symptoms, it is clear that the diagnosis provided by 2DE can be burdened by significant inaccuracy. Nevertheless, LVEF derived by 2DE remains the most used method in clinical practice because of its high availability and feasibility.

### 5.2. Real-Time Three-Dimensional Echocardiography

**Figure 1:**
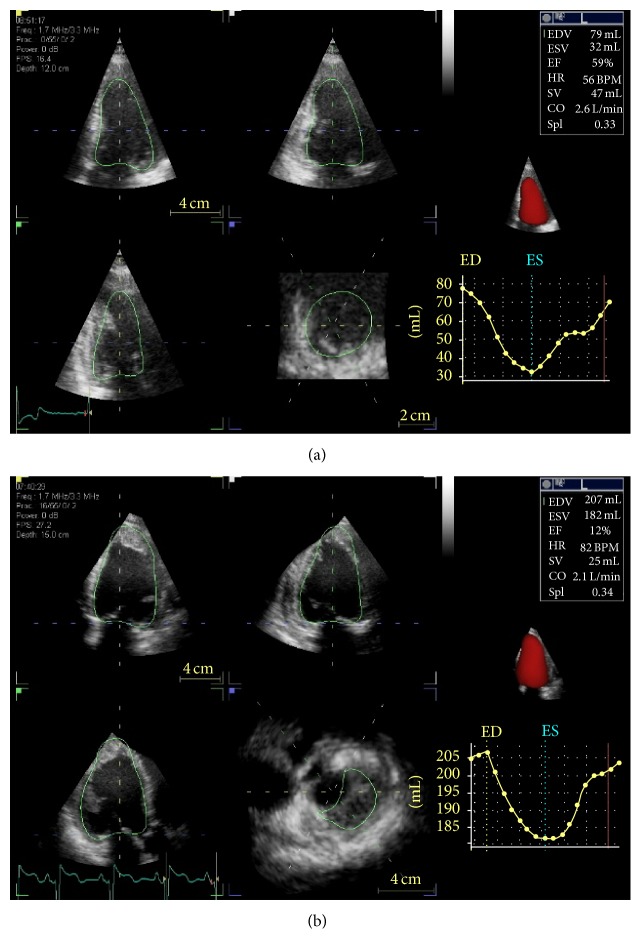
Three-dimensional echocardiography: evaluation of left ventricular ejection fraction in a normal patient (a) and in one with impaired function (b).

Real-time three-dimensional echocardiography can obtain a full-volume scan of the left ventricle, providing a quantification of volumes independently of geometrical assumptions. LVEF provided by RT-3DE (Figure 1) demonstrated elevated correlation with the values derived by cardiac magnetic resonance as shown in a study on 50 patients where Walker reported a correlation ranging from 0.90 to 0.97, while 2DE revealed a weak correlation (from 0.31 to 0.53) [15]. LVEF derived by RT-3DE showed the lower intraobserver and interobserver variability (0.017 and 0.027, resp.) and the best minimal detectable variation (4.8% intraobserver and 7.5% interobserver) [14].

### 5.3. Contrast Echocardiography

The accuracy in the measurement of volumes and LVEF is affected negatively by the poor quality of the acoustic window, which often limits the adequate visualization of the endocardial border. Use of contrast echocardiography demonstrated an incremental value, reducing the interobserver variability in evaluating the cardiac volumes and wall motion score index [16]. Use of contrast associated with 2DE resulted in a reduction of the interreader variability of LVEF from 14.3% (95% confidence interval, 11.7%–16.8%) to 8% (95% CI, 6.3%–9.7%; *P* < 0.001) [17]. Left ventricle opacization is recommended when two or more segments are not well visualized [18, 19]. The value of contrast administration with RT-3DE is uncertain; Hoffmann demonstrated a reduction of interobserver variability from 14.3% to 7.4% [17], while Thavendiranathan did not report any incremental value in comparison to noncontrast RT-3DE [14].

### 5.4. Nuclear Medicine Imaging

In the past, MUGA has been the most common alternative to echocardiography in the evaluation of chemotherapy-treated patients [20]. MUGA makes use of 99mTC-erythrocyte labeling enabling the visualization of the cardiac blood pool by *γ*-camera with electrocardiogram-triggered acquisitions. The final result provides a highly reproducible and precise quantification of LV volumes and dyssynchrony independently of geometrical assumption [21]. LVEF values provided by MUGA demonstrated reproducibility and sensitivity comparable to 3D echocardiography and CMR. Walker reported a correlation between LVEF evaluated by MUGA and CMR ranging from 0.87 to 0.97 [15]. Nevertheless, now MUGA is rarely used in clinical practice mainly because of the increased radiation exposure for patients and the introduction of new noninvasive techniques such as CMR and RT-3DE.

### 5.5. Cardiac Magnetic Resonance (CMR)

In the last years, CMR has emerged as the criterion standard technique in the evaluation of LV mass [22] and volumes. It provides a modeling of the cardiac chambers free from geometric assumptions and independently of acoustic window, providing the most accurate evaluation of global and regional myocardial dysfunction [23]. Armstrong demonstrated a decrease of LVEF and mass in a population of asymptomatic adult survivors of childhood cancer treated with anthracyclines in which other imaging techniques did not detect alterations [24] and similar findings have been reported by Ylänen [25]. CMR is indicated for the evaluation of patients treated with potentially cardiotoxic medications as an alternative to 2DE, particularly in patients with an echocardiographic cardiotoxicity diagnosis in whom the interruption of treatment could be inadvisable or in patients with poor echocardiographic images [26]. Although it has advantages, CMR usage is limited by its low availability and elevated cost. The method is not indicated in patients with metallic prosthesis, and the results are less accurate in subjects with arrhythmias.

## 6. New Methods and Strategies to Monitor Cardiac Function Independently of LVEF: Clinical Practice and Future Insights

### 6.1. 2DE and Tissue Doppler Imaging (TDI)

Alteration of diastolic function precedes the systolic dysfunction often representing the first sign of early cardiac dysfunction caused by anticancer agents [27]. 2DE is the best method for the evaluation of diastole. The decrease of the early to late ventricular filling velocities (*E/A*) ratio, the enlargement of the left atrium, and the increase of isovolumic relaxation time are common findings in chemotherapy-treated patients [28, 29] with impairment of diastolic function as well as reduction of* E* ^1^/*A* ^1^ ratio [5, 30, 31] and the increase of* E*/*E* ^1^ ratio >10 [28]. Although the diastolic dysfunction is frequent in chemotherapy-treated patients, its value in predicting the late development of cardiotoxicity is affected by many factors, such as aging, hypertension, and load conditions. Some authors reported that* E*,* E* ^1^,* E*/*A*, and isovolumic relaxation time did not predict late LVEF <50% within three years after the start of treatment [32]. Analysis of systolic function performed by TDI provided contrasting results: in a study by Fallah-Rad 42 patients demonstrated a significant reduction of lateral* S* ^1^ within three months from the start of chemotherapy. The decrease was ≥0.6 cm/s in all 10 patients who later developed LVD [33]. However, the result of the study was limited by several biases: above all, there was a high incidence of cardiotoxicity in a relatively small and young population. In effect, other studies failed in revealing a significant reduction of* S*′ in chemotherapy-treated patients [34, 35]. Myocardial deformation analysis derived from TDI demonstrated early alteration of both systolic and diastolic function after chemotherapy [36, 37]. Nevertheless, TDI measurements suffer from angle dependence, noise, translational movements, aliasing, and reverberation. For these reasons, myocardial deformation analysis derived by TDI has been almost totally replaced by speckle tracking echocardiography.

### 6.2. Two-Dimensional Speckle Tracking Echocardiography

**Figure 2:**
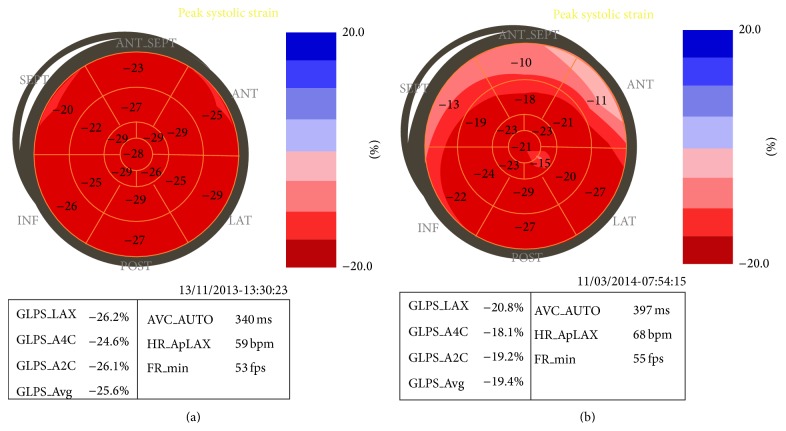
Bull's eyes showing a decrease of global and regional strain in a patient before (a) and after (b) treatment with chemotherapy. In the same patient the left ventricular ejection fraction was not significantly altered (see also Supplementary Videos 3 and 4).

Two-dimensional speckle tracking echocardiography (2D-STE) analyzes the myocardial deformation on two-dimensional images by tracking natural acoustic reflections and interference patterns, called “speckle.” The software is able to provide the percentage of distance variation (deformation) between speckles within a predefined region of interest, obtaining a value defined as “strain.” The velocity of the deformation is defined “strain rate.” 2D-STE provides an accurate definition of longitudinal, circumferential, and radial component of the ventricular deformation. Twist, untwist, and torsion are additional parameters that evaluate the torsional deformation of the left ventricle. Strain evaluated by 2D-STE detected early myocardial dysfunction in chemotherapy-treated patients [38, 39] (Figure 2 and Videos 3 and 4). The application of strain and strain rate to cardiotoxicity detection has been evaluated in several relatively small studies. Global longitudinal strain (GLS) appears to be the most sensitive parameter of deformation for the detection of early systolic dysfunction. Negishi demonstrated that, in 81 patients treated for breast cancer, GLS rate and early diastolic strain rate were significantly decreased at 6 months from treatment, in comparison to baseline value in 30% of patients who developed cardiotoxicity at 12 months. GLS percentage variation was the strongest predictor of cardiotoxicity (area under the curve, 0.84) and a reduction >11% was the optimal cut-off (sensitiveness 65%, specificity 94%) [40]. Similar results have been reported by Plana showing that a decrease of >9% in GLS after the third cycle of epirubicin was the best independent and accurate predictor of cardiotoxicity (sensitiveness 84%, specificity 80%; *P* = 0.0001) in a sample of cancer treated patients [26]. Stoodley showed a correlation between reduction of GLS and cumulative dose of anthracyclines [41]. Thavendiranathan [42] collected the fragmentary data from several studies and reported the results in a comprehensive, systematic review. The authors established that the percentage of change is a better indicator than a defined cut-off because of the variable baseline values. A variation in GLS ranging from 10% to 15% was the best predictor of future development of cardiotoxicity. Negishi established in 81 women treated with trastuzumab that GLS decrease can predict cardiotoxicity and an 11% reduction was the optimal cut-off (confidence interval 8.3%–14.6%) [40]. According to these findings, the recent consensus document released by the American Society of Echocardiography/European Association of Cardiovascular Imaging (ASE/EACVI) defined that a variation in GLS >15% is strongly predictive of future development of cardiotoxicity, while a variation <8% is not significant [26]. An important limitation associated with the use of STE is represented by differences in the deformation values provided by software from different vendors [43]. Waiting for a full standardization of the measurement, the recommendation is to evaluate the patients with the same software during the follow-up.

### 6.3. Three-Dimensional Speckle Tracking Echocardiography

Three-dimensional speckle tracking echocardiography (3D-STE) is one of the most advanced techniques in the evaluation of myocardial deformation. The possibility of evaluating the deformation on a full-volume model avoids the errors derived from the use of two-dimensional images. Xu compared 3D-STE to 2D-STE and revealed that GLS evaluation is slightly less feasible in comparison to 2D-STE (84.9% versus 97.2%); however, 3D-STE appeared less time-consuming (50.5 ± 6.4 sec versus 68.0 ± 9.2 sec) and the correlation was good between values obtained by the two methods appearing to be larger for structural measurements rather than for deformation analysis. Inter- and intraobserver variability ranged from 4.8% to 7.9% [44]. Yu demonstrated that childhood cancer survivors evaluated by 3D-STE had significantly reduced GLS and torsion (*P* < 0.001) and greater systolic dyssynchrony index in comparison to healthy controls [45]. Mornoş found that GLS evaluated by 3D-STE was superior to biomarkers and to LVEF in predicting future development of cardiotoxicity [46]. Although 3D-STE is a promising method, the studies which compared the technique to the other standard methods are few and included a small number of patients. A clear superiority to 2D-STE in predicting development of future cardiotoxicity has not yet been evaluated. Moreover, 3D-STE is not widely available in the echo-labs; thus its use, so far, has to be considered experimental.

### 6.4. Stress Echocardiography

Stress-echocardiography revealed contrasting results in the evaluation of chemotherapy-treated patients: some studies report a reduction of LVEF during stress in patients treated with chemotherapy in comparison to controls [47], while other studies did not report any incremental value of the technique [48, 49]. The only use of stress echocardiography is the evaluation of inducible ischemia in patients with high or intermediate pretest probability for coronary artery disease treated with drugs associated with ischemia (fluorouracil, bevacizumab, sorafenib, and sunitinib) [50].

### 6.5. Cardiac Magnetic Resonance

A good incremental value provided by CMR relies on the possibility of the method to perform a tissue characterization, identifying fibrosis and edema. The use of this technique can be used to investigate both early and late myocardial dysfunction in chemotherapy-treated patients.

#### 6.5.1. Detection of Early Cardiotoxicity

Preliminary human studies using T2 weighted sequences showed a significant increase of signal intensity after three days of therapy; this finding is indicative of interstitial edema and was predictive of LVEF reduction at 1 year [51]. A study of 22 patients receiving anthracyclines showed that, after three days of treatment, an increase >5 times of the ratio between signal intensity pre- and postcontrast administration was predictive of reduction of LVEF at 28 days and six months [52]. Delayed enhancement (DE) consists of the acquisition of delayed sequences after administration of gadolinium, which detects tissue with slow contrast washout, usually represented by scar or fibrosis. Fallah-Rad revealed subepicardial linear DE in the lateral wall of LV in all 10 patients with trastuzumab-induced cardiomyopathy even though, in only 40% of cases, DE during therapy was predictive of subsequent decline of LVEF [53]. A contrasting result has been recently presented by Drafts; the authors reported absence of DE during follow-up of anthracycline-treated patients, despite a significant decrease in LVEF [54].

#### 6.5.2. Detection of Late Cardiotoxicity

The improvement of cancer therapy has led to longer survival; accordingly, late cardiotoxic effects of chemotherapy have been observed in many patients. Reduction of LV mass has been evaluated as a marker of late cardiotoxicity. A sample of childhood cancer survivors presented LV mass <2 standard deviation (SD) of the mean value for normal population in 50% of cases [24]. A study carried out by Neilan to evaluate the prognostic value of CMR in adult patients revealed that LV mass index was an independent predictor associated with major adverse cardiovascular events [55].

### 6.6. Nuclear Medicine Imaging

Nuclear imaging is rapidly evolving, providing new techniques with potential involvement into the evaluation of chemotherapy-treated patients. Functional imaging techniques are able to assess pathophysiologic and neurophysiologic processes at the tissue level. Metaiodobenzylguanidine (MIBG) shares the same metabolic pathway as norepinephrine; when marked with 123I, it is able to represent a scintigraphic image of the efferent sympathetic nervous innervations of the heart. A decrease in myocardial uptake is a strong predictor of mortality and cardiac death [56]. Patients treated with anthracycline in a dose-dependent way showed a quick reduction in 123I MIBG uptake, which was predictive of late cardiotoxicity [57, 58]. A specific anti-myosin antibody marked with 111In has been used to identify cardiomyocyte injury and necrosis in patients treated with anthracyclines, representing a predictor of LVEF decrease [59]. Although these new techniques are very promising for the future, at the moment, their use remains limited to an experimental setting.

## 7. Conclusions

Use of chemotherapy and radiotherapy is essential for cancer patients and cardiotoxicity represents one of the most frequent causes of treatment interruption with significant implications on the prognosis. Early diagnosis and detection of high risk patients has become a central issue in the management of cancer patients involving both cardiologists and oncologists. Systematic and periodical monitoring of LVEF remains the most used technique to diagnose cardiotoxicity in clinical practice. 2DE is the most used method; however, 3DE has proved to be more accurate and reproducible and is preferable if available. CMR is the criterion standard but its low availability and the high cost limit its use to particular subsets of patients (poor acoustic window or patients in whom treatment interruption is highly hazardous). Nevertheless, the decrease of LVEF occurring only in end-stage has shown that it is not suitable as an early indicator of cardiotoxicity. Among the new techniques that evaluate the cardiac function independently of the analysis of volumes and only GLS derived by 2D-STE has validated supporting evidence in predicting late cardiotoxicity. Baseline and periodical evaluation of GLS is recommended by the recent guidelines by ASE/EACVI [26]. Promising techniques such as 3D-STE and tissue characterization performed by CMR are under investigation and could provide new insights into the future for the evaluation of chemotherapy-treated patients.

## Supplementary Material

Videos 1 and 2 show two-dimensional echocardiography. The videos show wall motion in the apical four-chamber view before (Video 1) and after (Video 2) one month of chemotherapy. The apical segments show a decreasing of the systolic function.
Videos 3 and 4 show two-dimensional echocardiography; The videos show wall motion in the apical four-chamber view before (Video 3) and after (Video 4) chemotherapy. left ventricular ejection fraction is not significantly altered, however, global longitudinal strain revealed a subclinical impairment of the deformation in this patient (see Figure 1).
